# Characterization and susceptibility of non-albicans *Candida* isolated from various clinical specimens in Lebanese hospitals

**DOI:** 10.3389/fpubh.2023.1115055

**Published:** 2023-03-10

**Authors:** Rola Husni, Maroun Bou Zerdan, Nadia Samaha, Mariana Helou, Youssef Mahfouz, Rim Saniour, Sawsan Hourani, Harout Kolanjian, Claude Afif, Eid Azar, Tamima El Jisr, Jacques Mokhbat, Emma Abboud, Rita Feghali, Edmond Abboud, Hiam Matta, Gilbert Karayakouboglo, Madonna Matar, Rima Moghnieh, Ziad Daoud

**Affiliations:** ^1^Lebanese American University, School of Medicine, Beirut, Lebanon; ^2^Department of Internal Medicine, Lebanese American University-Rizk Hospital, Beirut, Lebanon; ^3^Department of Internal Medicine, SUNY Upstate Medical University Hospital, Syracuse, NY, United States; ^4^Georgetown University School of Medicine, Washington, DC, United States; ^5^Faculty of Medicine and Medical Sciences, University of Balamand, Tripoli, Lebanon; ^6^Department of Internal Medicine, Saint George Hospital-University Medical Center, Beirut, Lebanon; ^7^Department of Laboratory, Makased General Hospital, Beirut, Lebanon; ^8^Department of Laboratory, Mount Liban Hospital, Hazmiyeh, Lebanon; ^9^Department of Laboratory, Rafic Hariri University Hospital, Beirut, Lebanon; ^10^Department of Laboratory, The Middle East Institute of Health University Hospital, Mount Lebanon, Lebanon; ^11^Saint Georges Ajaltoun Hospital, Ajaltoun, Lebanon; ^12^Haykal Hospital, Tripoli, Lebanon; ^13^Department of Internal Medicine, Notre Dame de Secours University Hospital, Byblos, Lebanon; ^14^College of Medicine, Central Michigan University, Saginaw, MI, United States; ^15^Department of Clinical Microbiology and Infection Prevention, Michigan Health Clinics, Saginaw, MI, United States

**Keywords:** fungal infection, non-albicans Candida, infection, microbiology, pathogens

## Abstract

**Background:**

Invasive fungal infections have presented a challenge in treatment. In the past, it was known that the frontrunner in such infections is *Candida albican*s with little emphasis placed on non-albicans *Candida* species (NAC). Studies worldwide have shown a rise in fungal infections attributed to non-albicans *Candida* species. The aim of this study is to describe the epidemiology of NAC infections along with an overview of resistance in Lebanese hospitals.

**Methods:**

This is a two-year observational multi-central descriptive study. Between September 2016 and May of 2018, a total of 1000 isolates were collected from 10 different hospitals distributed all over the country. For the culture, Sabouraud Dextrose Agar was used. Antifungal Susceptibility was evaluated by determining the Minimum Inhibitory Concentration (MIC) in broth (microdilution) of the different antifungal treatments.

**Results:**

Out of the 1000 collected isolates, *Candida glabrata*, being the most isolated species (40.8%), followed by *Candida tropicalis*: 231(23.1%), *Candida parapsilosis*: 103(10.3%), *a*nd other NAC species at lower percentage. Most of these isolates (88.67%) were susceptible to posaconazole, 98.22% were susceptible to micafungin, and 10% were susceptible to caspofungin.

**Conclusion:**

The change of etiology of fungal infections involving a significant increase in NAC cases is alarming due to the different antifungal susceptibility patterns and the lack of local guidelines to guide the treatment. In this context, proper identification of such organisms is of utmost importance. The data presented here can help in establishing guidelines for the treatment of candida infections to decrease morbidity and mortality. Future surveillance data are needed.

## 1. Introduction

The incidence and burden of fungal infections is rising globally. Fungal infections are a major concern for clinicians because it is associated with high morbidity and mortality, mainly in critical and immunocompromised patients. Serious and invasive *Candida* infections are usually hospital acquired. In the hospital setting, Non-albicans *Candida* species (NAC) are more frequently isolated (1).

Invasive candidiasis includes a variety of infectious conditions caused by *Candida* species. Invasive candidiasis is a serious infection that causes high mortality and morbidity. In the United States (US), around 25,000 cases of invasive candidiasis are reported annually (2). The most common and studied form of invasive candidiasis is candidemia, especially in intensive care patients (3). It remains a challenge to estimate the global incidence of candidemia and this is due to many factors including diagnostic techniques as well as the lack of surveillance systems for fungal infections (4). New diagnostic techniques are developing including Polymerase chain reaction and specific rapid antigen. Nevertheless, positive predictive values of non-culture techniques remain low while negative predictive values are high. Therefore, clinical suspicion of invasive fungal infections in combination with Candida diagnostics should be used in patients care. However, the reported annual incidence of candidemia in the US is around 9 cases per 100,000 (5). *Candida* species rank as the fourth most common cause of hospital-acquired bloodstream infections, after coagulase-negative *staphylococci* (CNS), *staphylococcus aureus*, and *enterococcus* spp. (6).

*Candida albicans* is the predominant isolate from patients with invasive candidiasis worldwide (7). However, a new threat has emerged over the last few decades, as NAC are increasingly recovered from patients. The most reported species of NAC include *C. glabrata, C. tropicalis, C. parapsilosis, and C. krusei* (8). Collectively along with *C. albicans*, these species are responsible for over 90% of the cases of invasive candidiasis (9). The frequency of each species varies with geographic differences in different countries (10–14), the local hospital epidemiology within the same country (15–17), the different units within the same hospital, underlying patient characteristics, and the antimicrobial treatment strategies and protocols (18, 19). Nevertheless, the clinical importance of NAC species lies in the potential antifungal resistance which can lead to treatment failure and its consequences.

Several studies (20–25) have estimated the incidence rates of candidemia in the Middle East and North Africa countries. Candidemia incidence rate was estimated to be the highest in Qatar, with a calculated rate of (15.4/100,000) (21) and the lowest in Iran (0.34/100,000) (20). In a study done by Koehler et al., European incidence of candidemia was estimated to be 79 cases per day, of which an estimated 29 patients might have fatal outcome at Day 30 (26). There was a higher proportion of *Candida* spp. other than *C. albicans* in the decade from 2010 till 2019 in population-based data (26).

Echinocandin and azole-resistance is increasingly reported in non-albicans *Candida* from cases of invasive candidiasis (27, 28). Exceptional resistance to antifungals in some Candida species, such as in Candida auris, constitutes a major threat to patients and has a significant impact worldwide. *Candida*'s ability to form biofilm represents a problem in the context of antifungal drug-resistance.

Lebanon is a small country in the Middle East Region where a prominent level of antimicrobial use has been documented (29). The current compiled antimicrobial susceptibility data have shed light on increasing bacterial resistance trends in this country, which were found to be comparable with data from some Eastern and Southern European countries (29). For that reason, it was important to understand the local epidemiology and subsequently to establish guidelines for the appropriate identification and treatment of such infections as well as for their prevention. This multicenter study aimed at describing the epidemiology and distribution of NAC species in the context of the global data, as well as identifying and determining the antifungal susceptibility profiles of 1000 NAC clinical specimens collected from various clinical infections.

## 2. Methods

### 2.1. Samples and study population

A total of 1,000 clinical samples including urine, vaginal swabs, sputum, blood, cerebrospinal fluid (CSF) and miscellaneous samples were collected prospectively from all patients having a positive fungal culture and presenting to 10 hospitals located in different geographic areas of the country between September 2016 and May of 2018 according to standard procedures. More than one clinical sample from the same patient with the same identification and same susceptibility profile were considered duplicates, and therefore only the first isolate was included. All clinical samples were inoculated on Sabouraud dextrose agar (Oxoid, Basingstoke, UK) to which 50 μg/ml of Gentamycin was added to suppress the growth of bacterial contaminants. Inoculated plates were incubated at 37° C for 72 hours aerobically, extended incubation was performed when needed. Isolates were identified by conventional methods using microscopic examination using KOH preparation, colonial morphology, and carbohydrate assimilation method using the API 20C Aux system (bioMerieux-Vitek, Hazelwood, Mo.).

### 2.2. Antifungal susceptibility testing

Antifungal Susceptibility testing was evaluated by determining the Minimum Inhibitory Concentration (MIC) in broth (microdilution method) of 7 different antifungals after 24 and 72 hours of incubation according to the CLSI M27 and M60 documents “Reference Method for Broth Dilution Antifungal Susceptibility Testing of Yeasts; Approved Standard—Second Edition- CLSI) (30) and re-analyzed according to CLSI M60 “Performance Standards for Antifungal Susceptibility Testing of Yeasts” after the second version of this document was issued (2020) (31). Each sample (*Candida* isolate) was run in duplicate to ensure accuracy of the results. The MICs were considered in Essential and Categorical agreement when their values fell within one dilution. When disagreement was observed, the experiment was repeated.

Antifungal standard reference powders were obtained commercially or directly from the drug manufacturer. After preparation, antifungal solutions were stored as recommended. All antifungal agents were assayed for standard units of activity. Antifungal solutions were standardized based on assays of the lots of antifungal powders.

Antifungal stock solutions were prepared at concentrations of at least 1280 μg/mL or ten times the highest concentration to be tested, whichever was greater.

The antifungal agents tested were: Amphotericin B, Micafungin, Caspofungin, Anidulafungin, Voriconazole, Fluconazole, and Posaconazole). Antifungal powders were dissolved depending on the chemical properties of each one. Some were dissolved in DMSO diluted in RPMI (Amphotericin B, Ketoconazole, Itraconazole, Posaconazole, Voriconazole). The concentrations to be tested were based on the breakpoint concentrations and the expected results for the quality control strains. Based on previous studies, the following drug concentration ranges were used: amphotericin B, 0.0313 to 16 μg/mL; flucytosine, 0.125 to 64 μg/mL; ketoconazole, 0.0313 to 16 μg/mL; itraconazole, 0.0313 to 16 μg/mL; fluconazole, 0.125 to 64 μg/mL; and new triazoles, 0.0313 to 16 μg/mL.

Quality control strains included *C. parapsilosis* ATCC 22019, *C. albicans* ATCC 90028, and *C. krusei* ATCC 6258. RPMI 1640 medium was used as a Synthetic Medium for susceptibility testing. Zwitterion buffers were used to buffer the media to a pH of 7.0 ± 0.1 at 25 °C. All organisms were sub-cultured from sterile vials onto Sabouraud Dextrose Agar.

### 2.3. Data analysis and interpretation

Patients' privacy and Identities were not revealed, all data were coded for that purpose. Statistical analysis was performed using SPSS version 20. Descriptive statistics such as frequency and percentage of *Candida* species were calculated.

### 2.4. Ethical clearance

All ethical deliberations and responsibilities were appropriately addressed, and the study was conducted after the approval of the Institutional Review Board (IRB) of the Lebanese American University. (IRB# LAU.SOM.RH1.26/Apr/2016).

## 3. Results

**Figure 1:**
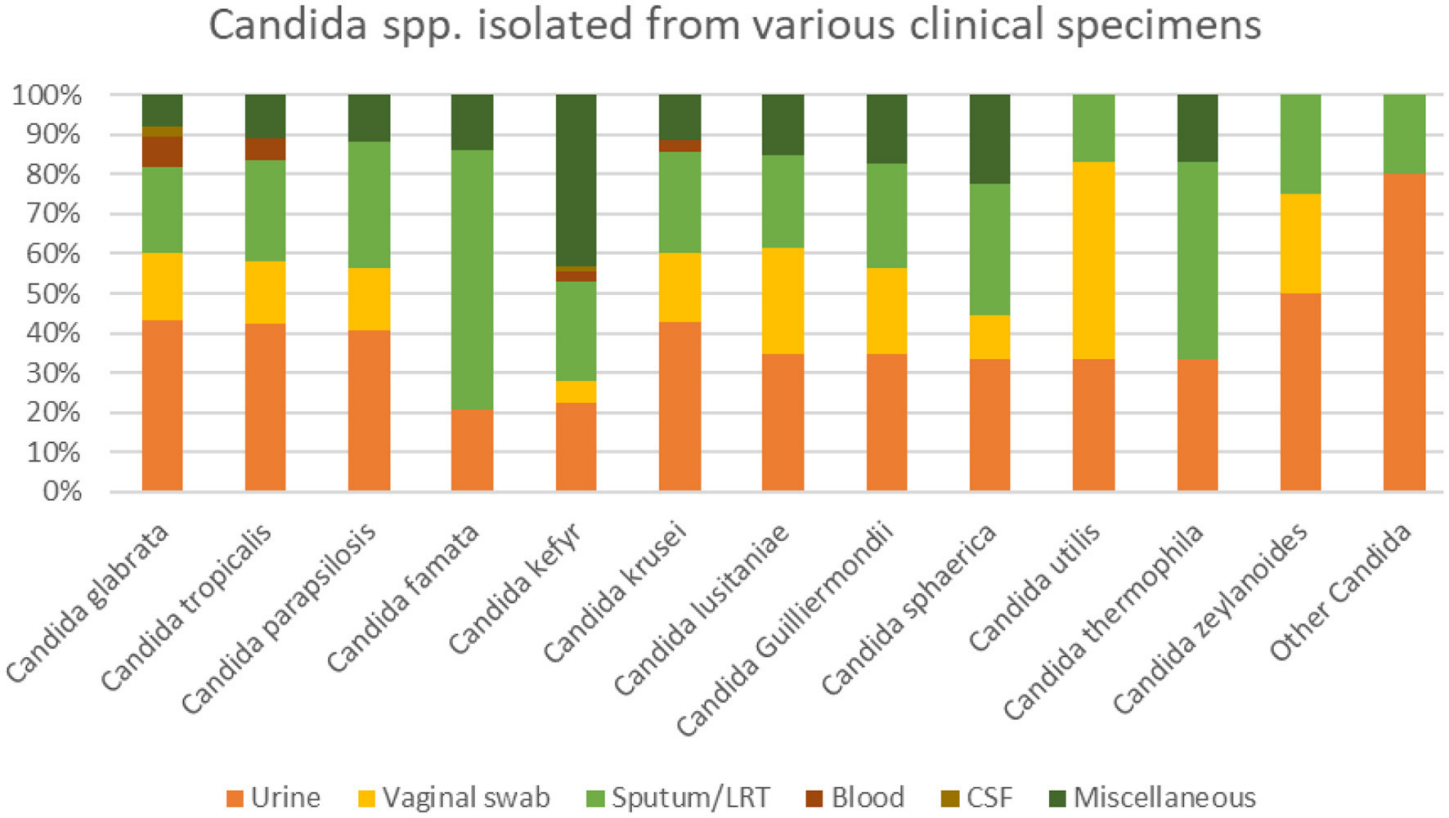
Candida spp. isolated from various clinical specimens.

A total of 1,000 yeast non-duplicates isolates were collected from different clinical samples (Figure 1). Among the isolates, 147 (14.7%) were recovered from vaginal swab, and 393 (39.3%) from urinary samples. The remaining 460 (46%) were isolated from sputa, blood, CSF, and miscellaneous sources. The distribution of *Candida* species was split between *Candida glabrata* (40.8%/ 408), *Candida tropicalis* (23.1%/ 231), *Candida parapsilosis* (10.3 %/ 103), *Candida famata* (7.2 %/ 72), *Candida kefyr (7.2 %/ 72), Candida krusei (*3.5%/ 35)*, Candida lusitaniae (*2.6%/ 26)*, and Candida guilliermondii (*2.3%/ 23). The remaining species were found to represent 3% of the total number of isolates found. The distribution of the isolates among the different hospitals are in Table 1.

**Table 1:** Distribution of the isolates among the different hospitals.

**Species**	**Number**	**MKH**	**MLH**	**MEH**	**HKH**	**SGA**	**RH**	**HNDS**	**HRH**	**SGUMC**
*Candida glabrata*	408	79	37	26	23	19	46	18	31	129
*Candida tropicalis*	231	41	21	17	15	11	26	10	15	75
*Candida parapsilosis*	103	20	13	12	6	3	9	5	8	27
*Candida famata*	72	11	7	8	0	7	7	7	10	15
*Candida krusei*	35	1	2	0	2	6	7	0	5	12
*Candida kefyr*	72	8	3	7	11	9	5	2	10	17
*Candida sphaerica*	9	0	1	0	1	2	0	1	2	2
*Candida zeylanoides*	4	0	0	0	1	0	0	0	1	2
*Candida lusitaniae*	26	8	2	0	0	1	1	2	1	11
*Candida utilis*	6	0	0	1	0	1	2	0	1	1
*Candida Guilliermondii*	23	5	3	4	0	1	2	0	1	7
*Candida thermophila*	6	1	1	0	0	0	1	0	2	1
Other Candida	5	0	1	1	0	1	0	1	0	1
Total	1000	174	91	76	59	61	106	46	87	300

^*^MKH, Al Makased Hospital, Beirut, Lebanon; MLH, Mount Lebanon Hospital, Mount Lebanon, Lebanon; MEH, Middle East Hospital, Mount Lebanon, Lebanon; HKH, Haykal Hospital, Tripoli, Lebanon; SGA, Saint Georges Ajaltoun Hospital, Keserwan, Lebanon; RH, LAU- Rizk Hospital, Beirut, Lebanon; HNDS, Hospital Notre Dame des Secours, Keserwan, Lebanon; HRH, Hariri Hospital, Beirut, Lebanon; SGUMC, Saint Georges University Medical Center, Beirut, Lebanon.

Among the 48 candidemia cases, 66.7 % had *C. glabrata*. Similarly, *C. glabrata* grew in 9 specimens among the 10 CSF specimens. Similarly, in the miscellaneous group (mostly abdominal and skin infections) the most common pathogens were *C. kefyr, Candida glabrata, C. tropicalis and C. parapsilosis* (Figure 1). *Candida auris* was not isolated in any of the specimen.

Susceptibility profile*:*

Table 2 shows Candida spp. Isolates susceptibility to various antifungals. *C. glabrata* isolates were highly 100% susceptible to Anidulafungin, and Amphotericin B, 98.5 % susceptible to micafungin, but none was susceptible to Fluconazole (Table 2). *C. tropicalis* isolates were 100% susceptible to Anidulafungin and Voriconazole and 99.6% to Amphotericin B. Only 4.3 % of *C. tropicalis* were susceptible to Fluconazole and 3.9 % to Pozaconazole. *C. parapsilosis* isolates were 100 % susceptible to Micafungin, Voriconazole, Anidulafungin and Amphotericin B. Only 6.8% were susceptible to Fluconazole and none to Pozaconazole. Multidrug resistance was not seen among any of the pathogens cultured. The data showed that the isolates found in blood and CSF were mostly C. Glabrata and C. tropicalis. These species had the highest pattern of resistance.

Table 2Candida spp. isolates susceptibility to various antifungals.

**Table:** 

***Candida* spp**.	**Antifungals (**μ**g/mL)**									
	**Ampho B**				**Micafungin**			**Caspofungin**		
	***n***	***Range***	**MIC50**	**MIC90**	***Range***	**MIC50**	**MIC90**	***Range***	**MIC50**	**MIC90**
**(A)**										
*Candida glabrata*	408	0.0156–1	0.19	0.5	0.015–0.25	0.016	0.031	0.0312–0.25	0.031	0.031
*Candida tropicalis*	231	0.0156–2	0.125	0.5	0.0156–0.25	0.031	0.05	0.0156–0.25	0.031	0.063
*Candida parapsilosis*	103	< 0.12–1	0.5	0.5	0.015–1	0.031	0.047	0.0312–0.5	0.047	0.063
*Candida famata*	72	0.004–0.06	0.015	0.015	0.03– 0.06	0.031	0.031	0.03–0.064	0.031	0.031
*Candida krusei*	35	0.047–1	0.25	0.32	0.047–0.25	0.031	0.25	0.094–0.25	0.03	0.1
*Candida kefyr*	72	0.12–2.0	0.25	1.25	≤ 0.008–0.03	0.015	0.015	≤ 0.008–0.03	0.015	0.015
*Candida sphaerica*	9	0.0156–1	ND	ND	0.015–0.031	ND	ND	0.015–0.031	ND	ND
*Candida zeylanoides*	4	0.5–1	ND	ND	0.015–0.031	ND	ND	0.015–0.031	ND	ND
*Candida lusitaniae*	26	1.5–8	2	4	0.0156–0.25	0.031	0.05	< 0.015–0.03	0.031	0.063
*Candida utilis*	6	0.015–0.125	ND	ND	0.015–0.6	ND	ND	0.015–0.6	ND	ND
*Candida Guilliermondii*	23	0.12–1	0.25	0.5	0.25–2	0.5	1	0.25–2	0.5	1
*Candida thermophila*	6	0.015–0.125	ND	ND	0.015–0.31	ND	ND	0.015–0.045	ND	ND
*Other Candida*	5	0.12–2.0	ND	ND	0.015–0.31	ND	ND	0.015–0.31	ND	ND
*Candida tropicalis*	0.0312–0.25	0.016	0.031	< 0.0156–0.6	0.016	0.031	0.22–>256	2	12	0.015–8
*Candida parapsilosis*	0.015–1	0.031	0.047	0.008–0.047	0.016	0.031	< 0.12–32	0.5	1.5	< 0.125–0.047
*Candida famata*	0.015–1	0.031	0.047	0.006–0.03	0.012	0.015	0.13–0.25	0.125	0.128	0.015–1
*Candida krusei*	0.047–0.25	0.094	0.25	0.047–0.25	0.094	0.25	64–128	64	128	0.25–0.5
*Candida kefyr*	0.015–0.12	0.03	0.06	≤ 0.015–0.03	0.015	0.03	0.12–0.5	0.125	0.25	≤ 0.015–0.03

**Table:** 

***Candida* spp**.	**Antifungals (**μ**g/mL)**											
	**Ampho B**					**Micafungin**					**Caspofungin**				
	***n***	***Range***	**MIC50**	**MIC90**	***Range***	**MIC50**	**MIC90**	***Range***	**MIC50**	**MIC90**		
**(B)**												
*Candida sphaerica*	0.015–0.0312	ND	ND	< 0.0156–0.5	ND	ND	0.12–0.5	ND	ND	0.015–0.031	ND	ND
*Candida zeylanoides*	0.015–0.03	ND	ND	0.015–0.03	ND	ND	4–Feb	ND	ND	0.06–0.25	ND	ND
*Candida lusitaniae*	0.015–0.6	0.031	0.063	0.015–0.6	0.031	0.063	0.125–32	2	6	0.015–0.6	0.031	0.047
*Candida utilis*	0.015–0.6	ND	ND	0.015–0.6	ND	ND	0.5–4	ND	ND	0.015–0.3	ND	ND
*Candida Guilliermondii*	0.25–2	0.5	1	0.032–0.13	0.03	0.06	0.75–1.5	0.89	1	0.032–0.13	0.03	0.06
*Candida thermophila*	0.015–0.31	ND	ND	0.015–0.31	ND	ND	0.5–1	ND	ND	0.015–0.31	ND	ND
*Other Candida*	0.015–0.31	ND	ND	0.015–0.31	ND	ND	0.015–0.31	ND	ND	0.015–0.31	ND	ND

IE, Insufficient Evidence that the organism or group is a good target for therapy with the agent, ND, Not Determined (for statistical significance purposes, MIC90 was not determined when the number of isolates was lower than 10.

## 4. Discussion

Fungi are increasingly recognized as important pathogens in critically ill and immunocompromised patients (32–36). The incidence of invasive candidiasis has increased over the past decade due to the increasing prevalence of immunosuppressive therapy, invasive surgical procedures, and use of indwelling medical devices (13). In addition, the increased use of broad-spectrum antibiotics leads to changes in the microbiome, shifting the balance toward fungi and more resistant strains of bacteria (37). Antifungal susceptibility is not uniform among different candida species, and some species are innately resistant while others acquire resistance to the first line of antifungals, Fluconazole and Echinocandins (38, 39). Because of this increase in resistance, candida speciation and Surveillance of Candida infection has become a must for every country as well as each hospital. Accordingly, the Clinical and Laboratory Standards Institute (CLSI) has recently adopted species-specific minimum inhibitory concentration (MIC) breakpoints for *Candida* species and recommends speciation and antifungal susceptibility of candida species isolated from sterile sites and causing invasive fungal infections. High rates of morbidity and mortality are associated with invasive Candida infections. The rate of mortality from candidemia is about 30%, while directly attributable mortality is between 19 and 24% (40, 41). Treating these infections requires antifungals that are expensive, and this is considered a burden in our country.

Table 3 summarizes the most common species in different countries around the world. Looking at the most common species in Lebanon, *C. tropicalis* was dominant in Lebanon with percentage ranging from 20 % to 45 % in some studies (56, 57). However, our study showed that *C. Glabrata* was the most common pathogen detected in all sites.

**Table 3:** Variation of Candida species among different countries.

**Study title**	**Authors et al**.	**Location of study**	**Duration of study**	**Most common Candida species**	**Resistance patters**
CDC: Invasive Candidiasis Statistics	CDC (42)	United States of America	Original article posted Jan 4, 2022?	95% of all invasive Candida caused by : *C. albicans, C. glabrata, C. parapsilosis, C. tropicalis, and C. krusei*. *C. albicans* is still the leading cause of candidemia in the United States, yet increasing proportions (two thirds) of cases by non-*albicans* species In some locations, *C. glabrata* is the most common species.	−7% of all *Candida* bloodstream isolates tested at CDC are resistant to fluconazole. - Echinocandin resistance seems to be rising, especially among *C. glabrata*
Prevalence of Non-Albicans Candida Infections in Women with Recurrent Vulvovaginal Symptomatology	Mintz, and Martens (43)	Jersey Shore Medical University, Neptune, USA	between July 2010 and February 2013	50% *C. albicans* and 50% NAC species. Across all visits: 60% *C. albicans*, 56.7% NAC, and 16.7% both a *C. albicans* and a NAC species. Among all isolated NAC species: 28.6% *C. glabrata*, 23.8% *C. krusei*, 23.8% *C. parapsilosis*, and 23.8% other *Candida* species	
Epidemiology of candidemia at a tertiary Canadian hospital, 2004–2013	Remington et al. (44)	Edmonton, Canada	2004-2013	*C. albicans* 48.0% *C. glabrata* 32.0% *C. parapsilosis* 5.2% *C. tropicali* (4.0 % *C. krusei* 4.0% *C. lusitaniae* 1.6 % *C. kefyr* 1.2%, *C. guilliermondii* 0.8%, and 1 unknown *Candida* species	-Fluconazole: 4.5 % resistance in *C. albicans* 8.3 % resistance in *C. parapsilosis*-Voriconazole: 0.9 % resistance in *C. albicans* 16.7 % resistance in *C. parapsilosis* 26.6 % resistance in *C. glabrata*-Caspofungin: 15.3 % resistance in *C. albicans* 95 % resistance in *C. glabrata*-Amphotericin B: 0% resistance in all species tested
Epidemiology of Candidemia: Three-Year Results from a Croatian Tertiary Care Hospital	Marekovic et al. (45)	Croatia	2018-2020	*Candida albicans* (43.53%) *C. parapsilosis* (31.76%) *C. glabrata* (12.36%) *C. krusei* (5.29%) *C. tropicalis* (2.35%) *C. lusitaniae* (2.35%)	-Fluconazole resistance: *C. albicans* 3.92 %, *C. parapsilosis* 83.33 %, *C. glabrata* 28.57 % -Andilofungin resistance: *C. albicans* 1.96 %, *C. parapsilosis* 2.78 %, *C. glabrata* 0.0 % -Caspofungin, Amphotericin B resistance: *C. albicans* 5.88 & 0.0 %, *C. parapsilosis* 0.0 & 0.0%, *C. glabrata* 0.0 & 0.0 %, respectively
Increasing Incidence and Shifting Epidemiology of Candidemia in Greece: Results from the First Nationwide 10-Year Survey	Mamali et al. (46)	Greece	2008-2018	*C. parapsilosis* species complex (SC) (41%) *C. albicans* (37%) *C. glabrata SC* (10%) *C. tropicalis* (7%) *C. krusei* (1%) Other rare Candida spp. (4%).	-Fluconazole resistance: *C. albicans* 3%, *C. parapsilosis* 20%, *C. glabrata* 5%, *C. tropicalis* 6% -Voriconazole resistance: *C. albicans* 3%, *C. parapsilosis* 1%, *C. glabrata* N/A, *C. tropicalis* 1% -Caspofungin: *C. albicans* 3%, *C. parapsilosis* 0%, *C. glabrata* 2%, *C. tropicalis* 2%, C. krusei 3%
Epidemiology of candidemia in NICE area, France: A five-year study of antifungal susceptibility and mortality	Vannini et al. (47)	Nice, France	January 2014 to December 2018	*C. albicans* (44%) *C. glabrata* (22%) *C. parapsilosis* (13%). Non-albicans Candida decreased from 68% in 2014 to 45% in 2018	All *C. albicans* and *C. parapsilosis* isolates were susceptible to fluconazole, caspofungin, voriconazole and amphotericin B
Changes in the epidemiological landscape of invasive candidiasis	Lamoth et al. (48)	Global	Published 03 January 2018	over the last decade, there's been a decrease in the proportion of *C. albicans* and an increase in *C. glabrata* and *C. parapsilosis*. **USA:** the proportion of *C. albicans* has dropped significantly and it now accounts for < 50% of *Candida* infections. The largest proportional increase in the USA is in *C. glabrata*, which now accounts for one-third or more of all candidemia isolates. This is followed closely by an increase in *C. parapsilosis*, which accounts for ~15% of all isolates. **Australia**: between 2004 and 2015 *C. glabrata* increased from 16% to 27% of all isolates. **Denmark**: *C. glabrata* accounted for 26% of isolates by 2009, like the 27% seen in a multicentre study in Belgium. **Scotland**: *C. glabrata* accounts for 21% of isolates, but in Spain *C. glabrata* only 13%, third behind *C. albicans* and *C. parapsilosis*. **Norway**. *glabrata* accounts for only 15% of the isolates but is still ranked second behind *C. albicans*, which made up 68% of all *Candida* isolates. **Latin America and Africa** :predominant species are *C. albicans* and *C. parapsilosis*. **Brazil** : surveillance from 16 hospitals revealed *C. albicans* (34%), *C. parapsilosis* (24%) and *C. tropicalis* (15%) as the predominant species, numbers that are like earlier surveillance data in 11 centers from nine cities: *C. albicans* (41%), *C. parapsilosis* (21%) and *C. tropicalis* (21%). **Latin America**: seven-country, 20-center surveillance study showed *C. albicans* (38%) and *C. parapsilosis* (27%) were predominant, and a 10-center study, where again *C. albicans* (44%) and *C. parapsilosis* (26%) were predominant. **South Africa**: *C. albicans* and *C. parapsilosis* are predominant, but data are dependent on whether the hospitals are private or public. In public hospitals it is *C. albicans* (46%) and *C. parapsilosis* (35%), while in private sector hospitals it is *C. parapsilosis* (53%) and then *C. albicans*. **Asia Pacific**: seven-country, 13-hospital study showed C. *albicans* was most common (36%) but *C. tropicalis* was second (31%). **Taiwan**: increasing *C. glabrata* rates, with *C. glabrata* going from 1.1% in 2003 to 21.6% in 2012. **India and Pakistan**: *C. tropicalis* is the most prevalent species, followed by *C. albicans*. In Pakistani adults, *C. albicans* (12%) was fourth most prevalent following *C. tropicalis* (38%), *Parapsilosis* (18%) and *C. glabrata* (16%)	-Fluconazole: Resistance rates in the **USA** for *C. albicans, C. tropicalis* and *C. parapsilosis* are 2%, 5% and 4%, respectively. These are like rates found in **Norway and Switzerland**. 10% of *C. glabrata* are resistant to fluconazole in the **USA**, like rates in **Belgium and Australia**. Fluconazole resistance in *C. tropicalis* are higher in **Taiwan, Australia, and Belgium** than in the **USA**.
Epidemiology, risk factors and outcomes of Candida albicans vs. non-albicans candidemia in adult patients in Northeast China	Zhang et al. (49)	Shenyang, Northeast China	2012-2017	*C. parapsilosis* 38.3% *C. albicans* 35.6% *C. glabrata* 13.9% *C. tropicalis* 10% *C. krusei* 1.1% *C. famata* 1.1%	-Fluconazole resistance : 6.7 % including *C.albicans* (3.1%), *C. parapsilosis* (2.9%), *C. tropicalis* (33.3%) and both isolates of *C. krusei*. -Voriconazole resistance: (5.6%) -Amphotericin B: except for one isolate of *C. glabrata*, all other *Candida* isolates were susceptible
Changing epidemiology of non-albicans candidemia in Korea	Ko et al. (50)	Korea	6 years	increase of *C. glabrata* (from 21.3% to 28.5%) and a decrease of *C. parapsilosis* (from 36.5% to 24.7%) were noticed. During the study period, *C. tropicalis* (36.4%) was most common NAC followed by *C. glabrata* (28.5%), *C. parapsilosis* (24.7%), and *C. krusei* (2.6%)	Replacement of primary amphotericin B treatment with echinocandins decreased amphotericin B resistance from 7.8% in 2011 to 0% in 2014
Antifungal Resistance of Candida Species in Bacolod City, Philippines	Juayang et al. (51)	Bacolod City, Philippines	from July 2017 to July 2018	*C. albicans* (62%) *C. tropicalis* (15%) *C. cefirrii* complex (10%)	-Voriconazole: *C. albicans* 7.4 % resistant, NAC 10% resistant -Fluconazole: *C. albicans* 3.7 % resistant, NAC 5.6% resistant−5-Fluorocytosine: *C.albicans* 2.8 % resistant, NAC 29 % resistant -Caspofungin: 0% resistance found across all species tested
Non-albicans Candida species: Emergence of neglected pathogens among population of Karachi	Jabeen et al. (52)	Karachi, Pakistan	October 2016-September 2017	predominance of *C. albicans* (54.5%) over non- albicans Candida species (45.5%). NAC: *C.glabrata* (16.7%) *C.tropicalis* (16.5%) *C. rugosa* (3.8%) *C.krusei* (3.9*%), C*. *parapsilosis* (1.4%) *C. guilliermondii* (1.4%), *C. kefyr* (0.9%), *C. zeylanoides* (0.5%), *C. apicola* (0.2%) and *C*. *lipolytica* (0.2%)	
The epidemiology of Candida species in the Middle East and North Africa	Ghazi et al. (53)	Middle East and North Africa (MENA)	ND	*C. tropicalis* prevails in Saudi Arabia, Lebanon, and UAE, *C. parapsilosis* is the most common species in Kuwait, Egypt, and Turkey	
Changing trends in epidemiology and antifungal susceptibility patterns of six bloodstream Candida species isolates over a 12-year period in Kuwait	Khan et al. (54)	Kuwait	2006–2017	*C*. *albicans* (37.22%) *C*. *parapsilosis* (34.67%) complex isolates including *C*. *orthopsilosis* (n = 5) and *C*. *metapsilosis* (n = 2) *C*. *tropicalis* (14.5%) *C*. *glabrata* (10.22%) *C*. *krusei* (1.81%) *C*. *dubliniensis* (1.5%). There was an overall increase of 8.8% candidemia cases caused by non-*albicans Candida* species during 2012–2017. between 2006–2011 and 2012–2017: *C*. *albicans* 41.8% and 33.1%, *C*. *parapsilosis* complex 32.01% and	-Fluconazole: 3/371 *C*. *albicans* isolates during 2006–2011 and 5/363 during 2012–2017 were resistant to fluconazole. 70.1% *C*. *albicans* isolates were susceptible to fluconazole during 2006–2011 compared to 58.1% during 2012–2017 1/310 *C*. *parapsilosis* isolates during 2006–2011 and 21/446 during 2012–2017 were resistant. 98.0% of
				37.04%, *C*. *tropicalis* 13.59% and 15.31%, and *C*. *glabrata* 8.77% and 11.51%, and *C*. *krusei* 2.0% and 1.7%, respectively. although *C*. *albicans* was the most frequently isolated species during 2006 to 2012, it was replaced by *C*. *parapsilosis* sensu stricto in the next four years (2013 to 2016)	*C*. *parapsilosis* isolates were susceptible during 2006–2011 as compared to 93.4% during 2012–2017
Prevalence and species distribution of Candida bloodstream infection in children and adults in two teaching university hospitals in Egypt: first report of Candida kefyr	Reda et al. (55)	Cairo, Egypt	2019-2020	Among Adults: *C. Albicans*: 28% *C. Non Albicans*: 72%, among which: *C. tropicalis* 27.8% *C. parapsilosis* and *C. glabrata* 16.7, 11.1% respectively. Only one *C. lusitaniae, C. utilis*, and *C. kefyr* (5.5%) were detected in adults. The uncommon *Candida*, which was *Candida* species other than *C. albicans, C. parapsilosis, C. tropicalis, C. glabrata*, and *C. krusei*, represented 16.6% of all candidemia In pediatrics: *C. albicans* 48.3% while non-albicans 51.6%. Of the NAC, most common were *C. tropicalis* (22.5%), *C. parapsilosis* (10.8%), *C. lusitaniae* (6.4%), *C. krusei* (4.3%), *C. famata* (4.3%), and *C. utilis* (2.2%). One *C. kefyr (*1.1%) was also isolated from pediatric patients. The uncommon *Candida* species represented 14% of all candidemia	
Epidemiology and burden of invasive fungal infections in the countries of the Arab League	Kmeid et al. (56)	Database Search	Arab League Countries	*C. albicans* is still the most com- monly isolated species in blood in the Arab League countries. 2015(2009-2014) **Qatar**: *C. Albicans* (38.7%) *C. Tropicalis* (18.9%) *C. Glabrata* (16.3%) *C. Parapsilosis* (12.6%) *C. Krusei* 9 (1.4%) **Algeri**a (2004-2014): *Parapsilosis* (36.6 %) *C.albicans* 31.6% *C. Tropicalis* 23.3% *C. Krusei* 3.3% Lebanon: 9-year study published in 2015: *C. Albicans* (24.7%) *C. Tropicalis* (34-45%) *C. Glabrata* (25-36%) *C. Parapsilosis* (9-22 %) *C. Krusei* 9 (5-11%)	Methods of testing varied widely between studies -Fluconazole: *C. albicans* susceptibility ranged from 38.5 % and 96.2 %. *C. tropicalis* 11.1%-100% susceptible, *C. glabrata* 50%-94.2% susceptible, and *C. parapsilosis* 66.7%-100% susceptible. -Voriconazole: *C. albicans* susceptibility was between 94-100%, *C. tropicalis* 83%-100%, *C. glabrata* 74%-100%, and *C. parapsilosis* 100%. - Caspofungin: 81-100% *Candida* susceptibility -Amphotericin: 90-100% susceptibility
Update on invasive fungal infections in the Middle Eastern and North African region	Osman et al. (57)	Lebanon and KSA	2011-2012	*C. albicans* (56%) *C. tropicalis* (20%) *C. glabrata* (14%)	
-	-	KSA	August 2012 and May 2016	*C. albicans (38.3%)* *C. tropicalis (16.7%)* *C. glabrata (16%)* *C. parapsilosis (13.6%)*	
-	-	Kuwait	2014-2016	*C. albicans* (32%) *C. parapsilosis* (32%) *C. tropicalis* (20%) *C. glabrata* (13%) *C. dubliniensis* (1%) *C. famata* (1%) *C. auris* (1%)	
-	-	Turkey	2010-2016	*C. albicans (48.1%)* *C. parapsilosis (25.1%)* *C. glabrata (11.7%)*	
Ten-Year Review of Invasive Candida Infections in a Tertiary Care Center in Saudi Arabia	Omrani et al. (58)	Saudi Arabia	January 2003-December 2012	*C. Albicans* 38.7 % *C. Tropicalis* 18.9 % *C. Glabrata* 16.3 %	
Comparative Analysis of Candida Albicans Vs. Candida Non-Albicans Infection Among Pediatric Patients at King Abdulaziz University Hospital	Al-Sofyani et al. (59)	Saudi Arabia	March 2018 to February 2020	*C. albicans*: 37.7 % *C. non-albicans*: 62.3 % Among non-albicans Candida: *Candida parapsilosis*: 24.6% *Candida topicalis*: 19.7% *Candida glabrata*: 6.6%.	
Epidemiology and antifungal susceptibility testing of non-albicans Candida species colonizing mucosae of HIV-infected patients in Yaoundé (Cameroon)	Ngouana et al. (60)	Yaoundé, Republic of Cameroon	January 2012 to October 2013	(37.2%) C. albicans (0.7%) C. Africana (56.6%) NAC isolates. The NAC isolates were grouped into 13 species including: C. krusei (18.1%) C. glabrata (10.9%) C. tropicalis (8.5%) a C. parapsilosis (5.9%)	-Amphotericin B and itraconazole: All the isolates appeared to be wild-type -Fluconazole: One (1/33) isolate of *C. glabrata* was resistant. *C. parapsilosis* isolates appeared all susceptible to fluconazole. *C. tropicalis* showed 50% resistance to fluconazole.
Distribution of Candida albicans and non-albicans Candida species isolated in different clinical samples and their *in vitro* antifungal susceptibility profile in Ethiopia	Seyoum at al. (61)	Ethiopia	January 2018 to September 2018	*C. albicans* 49.8 % Non *albicans Candida* species 43.1 % Other yeasts 7.2 % Among NAC species: *C. krusei* 15.6% *C. famata* 14.4% *C. rugosa* 11.1% *C. lusitaniae* 10.0%	-Fluconazole: 85.6, 3.9, and 10.5% of the isolates were susceptible, intermediate, and resistant, respectively, regardless of species. *C krusei* was 100% resistant -Voriconazole: 99.4% of *Candida* isolates were susceptible -Caspofungin and micafungin: 4 % resistance -Flucytosine: 86.2, 6.6, and 7.2% were susceptibility, resistant, and intermediate, respectively
Prevalence and Speciation of Non-albican Vulvovaginal Candidiasis in Zaria	Jimoh et al. (62)	Zaria, Nigeria	February 2012 to March 2013	60.7% *Candida parapsilosis* 21.4% *Candida tropicalis* 17.9% *Candida* *glabrata*.	
Non albicans Candida species: A review of epidemiology, pathogenicity and antifungal resistance.	Deorukhkar & Saini (63)	Database Search	Published in 2015	*C. tropicalis*: the most common NAC spp. from HIV infected patients with oropharyngeal candidiasis (OPC) *C. glabrata* : 2nd or 3rd most common Candida spp. isolated from various types of candidiasis. *C. parapsilosis* : one of the important causes of systemic candidiasis in neonates and ICU patients. *C. krusei*: causes disseminated infections in bone marrow or stem cell transplant recipients and hematological malignancy patients	
Species distribution and antifungal susceptibility patterns of clinical Candida isolates in North Lebanon: A pilot cross-sectional multicentric study	Osman et al. (64)	Medical centers in North Lebanon	January 2014-August 2019	Non-albicans Candida (NAC) constituted 68.8% of the isolates *Candida glabrata* was predominant followed by *C. parapsilosis, Candida tropicalis*.	NAC species are intrinsically less-susceptible to the most commonly used anti- fungals especially fluconazole and echinocandins. *C. glabrata* was found to be 88.9% susc to Ampho B, none to Fluconazole, 83.3% to Itraconazole, 67% to Voriconazole.

In a study done in one region in Lebanon on 93 Candida isolates, *C. glabrata* was the most common, followed by *C. parapsilosis*, and *C. tropicalis* which is similar to our results (64).

While *C. tropicalis* and *C. parapsilosis* are the most common species found in many countries with variable percentages in African countries: Nigeria: *C. parapsilosis* 60.7% and *C. tropicalis* 21.4 % (62), Algeria: *C. parapsilosis* 36.6% and *C. tropicalis* 23.3 % (56), Cairo: *C. parapsilosis* 16.7% and *C. tropicalis* 27.8 % (55), South Africa *C. parapsilosis* 35% (48). Similar percentages are also seen in South America *C. parapsilosis* 24% and *C. tropicalis* 15 % (48) and the Middle East and Arab countries; Saudi Arabia: *C. parapsilosis* 13.6% and *C. tropicalis* 16.7 % (57), Kuwait: *C. parapsilosis* 32 to 34 % and *C. tropicalis* 14.5 to 20% (54, 57), Turkey: *C. parapsilosis* 25.1% (57) and Qatar: *C. parapsilosis* 12.6% and *C. tropicalis* 18.9 % (56). In Europe, some countries have similar percentages with *C. parapsilosis* like Greece 41 % (46). Thus, understanding the local epidemiology of resistance of NAC and their susceptibility profiles provided by our data has an important role in guiding care of patients with the adequate choice of antifungal.

Invasive Candidiasis is a major healthcare problem associated with high mortality and cost. According to the country's susceptibility pattern described above, non-albicans species are increasing and are associated with reduced antifungal susceptibility. Thus, Echinocandins are the drug of choice in empirical treatment for these patients with risk factors for invasive candida infection. However, according to the literature de-escalation and the use of oral therapy are acceptable strategies to follow in the management of such patients. Voriconazole is also an acceptable alternative if the patient did not receive prior azoles therapy whether prophylaxis or therapeutic. Clearly, this data sheds light on proper management of patients with fungal infections. However, patients with vaginal infection who have *C. glabrata* need further studies and consideration of treatment since oral medications might not be the best choice as seen in our data. In addition, CNS infections should be treated with amphoteric B not Echinocandins because of lack of concentration in the CNS (65).

Newer technologies such as Maldi-tof-MS and molecular techniques are considered the most reliable for microbial identification. However, sugar fermentation-based techniques are still reliable and commonly used for yeast identification. In a study by Arastehfar (66), API 20C AUX correctly identified 83.7% of yeast isolates. Another study Using sequencing as a standard technique for NAC identification, 78.9% of the isolates were correctly identified by API 20C AUX while the Vitek 2 YST ID Card system yielded 71.8% and Bruker and Vitek proteomic techniques yielded 90.1% and 80.3% of correct identification (67). These studies, in addition to many others, show a high accuracy of yeast identification of sugar fermentation-based methods and support their use for yeast identification.

Invasive Candida infections has high mortality and the yield of culture remains low. Mucocutaneaous Candida infection and colonization have a high positive predictive correlation with invasive infection. Thus, any patient with risk factors of invasive candidiasis should be empirically or preemptively treated before susceptibility pattern in determined. This is why it is important to know the epidemiology and resistance patterns in order to direct our treatment properly especially in the ICU and in immunocompromised patients.

The importance of such studies is obvious. It can help in establishing guidelines of treatment for such infections. However, this should be complemented by continuous proper surveillance system to interpret the dynamic changes of the epidemiology. For example, it is important to note that lately *Candida auris* was reported in one of the tertiary centers in our country but not in others. Moreover, further studies about the epidemiology from animals and environmental candida species are needed as part of the One Health approach to decrease morbidity and mortality associated with this infection.

## Data availability statement

The raw data supporting the conclusions of this article will be made available by the authors, without undue reservation.

## Ethics statement

The studies involving human participants were reviewed and approved by the Institutional Review Board (IRB) of the Lebanese American University (IRB# LAU.SOM.RH1.26/Apr/2016). Written informed consent for participation was not required for this study in accordance with the national legislation and the institutional requirements.

## Author contributions

All authors listed have made a substantial, direct, and intellectual contribution to the work and approved it for publication.
